# Association between education and health outcomes among adults with disabilities: evidence from Shanghai, China

**DOI:** 10.7717/peerj.6382

**Published:** 2019-02-19

**Authors:** Tong Ge, Qi Zhang, Jun Lu, Gang Chen, Mei Sun, Xiaohong Li

**Affiliations:** 1 Department of Health Policy and Management/ School of Public Health, Fudan University, Shanghai, China; 2 China Research Center on Disability Issues at Fudan University, Fudan University, Shanghai, China; 3 Key Laboratory of Health Technology Assessment, National Health and Family Planning Committee (Fudan University), Fudan University, Shanghai, China; 4 School of Community and Environmental Health, Old Dominion University, Norfolk, United States of America; 5 Department of Health Law and Health Inspection/ School of Public Health, Fudan University, Shanghai, China

**Keywords:** Education, Health, Disability, China, Health examination

## Abstract

**Background:**

Adults with disabilities often have worse health outcomes than do their peers without disabilities. While education is a key determinant of health, there is little research available on the health disparities across education levels among adults with disabilities in developing countries. We therefore examined the association between health outcomes and education among adults with disabilities in Shanghai, China.

**Methods:**

We used the health examination records of 42,715 adults with disabilities in Shanghai in 2014. Five health outcomes, including two diseases (fatty liver and hemorrhoids) and three risk factors (overweight [body mass index ≥ 24]), high blood glucose, and high blood lipid), were evaluated. Descriptive statistics and Pearson’s chi-square test were used to assess differences in participants’ demographic and disability characteristics. Pearson’s chi-square test and Fisher’s exact test were conducted to compare the prevalence of each health outcome among the different education levels. Finally, logistic regression analyses were conducted to explore the association between education and health outcomes after adjusting for sociodemographic characteristics.

**Results:**

People with an elementary school or lower degree had the highest prevalence of overweight (52.1%) and high blood glucose (20.8%), but the lowest prevalence of hemorrhoids (18.6%) and fatty liver (38.9%). We observed significant differences in the association between education and health outcomes across disability types. For example, in physically disabled adults, higher education was related to higher odds of hemorrhoids (*p* < 0.001); however, there were no significant disparities in hemorrhoids across the education levels among adults with intellectual disabilities.

**Discussion:**

Compared with people without disabilities, adults with disabilities in Shanghai have relatively poor health. The association between education and health outcomes differed according to the health condition and disability type. To reduce the prevalence rate of overweight and high blood glucose among people with disabilities, tailored health promotion initiatives must be developed for people with lower education levels. In contrast, specific attention should be paid to the prevention of hemorrhoids and fatty liver among more-educated people with disabilities. Our study provides important evidence for targeting educational groups with specific disability types for health promotion and intervention.

## Introduction

The World Report on Disability has revealed that more than one billion people worldwide—about 15% of the total global population—live with some kind of disability (World Health Organization, 2011). The number of people with disability will continue to rise as populations age, alongside the aggravation of chronic health conditions (World Health Organization, 2015; Cieza et al., 2018). Because of this enormous population and the huge associated burden, disability is increasingly being regarded as a global public health burden, human rights issue, and societal priority (Troeger et al., 2018; Ramsey, Svider & Folbe, 2018).

Individuals with disabilities tend to have poorer health, achieve lower education levels, have fewer economic opportunities, and have a higher likelihood of poverty than do individuals without disabilities. For example, individuals with mental disabilities have a risk of preventable death four to six times that of the general population (Harris & Barraclough, 1998). Moreover, individuals with disabilities are three times as likely not to have access to health care and four times as likely to be treated poorly by the health care system (Bickenbach, 2011). Disabilities, together with diverse barriers, can impede people’s full and active involvement in daily life and social activities. Individuals with disabilities also often have unfulfilled health and rehabilitation needs and encounter difficulties in accessing mainstream health care services, which has in part led to their overall poorer health status and position as one of the most socially excluded populations in any society (Kirschner, Breslin & Iezzoni, 2007).

Education is a significant determinant of health (Winkleby et al., 1992). A positive correlation between education and health is an empirical regularity in the disciplines of public health, health education and health literacy studies, sociology, health economics, and in various multidisciplinary approaches (Higgins, Lavin & Metcalfe, 2008; Gase et al., 2017; Ross & Wu, 1995; Grossman, 2008; Han, 2017; Sen, 2002). Each discipline has explained this correlation in a different way; in health economics, a higher level of schooling is related to the potential to earn a higher income, and thereby afford better health care (Albert & Davia, 2011). In sociology, education is regarded as a source of socio-psychological resources, which protect against the factors detrimental to health (Ross & Wu, 1995). In public health, individuals with higher terminal education are considered to be more likely to choose healthy habits (Rahkonen, Berg & Puska, 1995). Therefore, the positive association between education and health can last for a lifetime after individuals complete their terminal degrees. Despite extensive research focused on the association between education and health among people without disabilities, few studies have examined the association in the disabled population, especially in developing countries. Without adequate information about the effects of education on health outcomes in this population, it is difficult to implement effective interventions to improve the health and quality of life of people with disabilities (Tomlinson et al., 2009).

China has more than 1.3 billion people—about one-fifth of the world’s population. According to the Sixth China National Census and the Second China National Sample Survey on Disability, around 85.02 million Chinese people live with some form of disability (China Disabled Persons’ Federation, 2015). The impact of western educational reforms, natural disasters, and more participation in the global economy has provided incentives for the Chinese government to start developing strategies and policies to satisfy the needs of individuals with disabilities (Zhang & Spencer, 2015). For example, in 2017 the Regulations on Education for Persons with Disabilities were revised to vigorously promote inclusive and special education, including “installing education in the home” and establishing distance education for students who cannot go to regular schools (The State Council of the People’s Republic of China, 2017). In spite of these positive changes, the health status of different educational groups in the disabled population of China remains unclear.

Shanghai is the most developed city in China; it has a population of around 24.15 million and approximately 423,520 disabled people (Shanghai Bureau of Statistics, 2016). The Shanghai Disabled Persons’ Federation (SHDPF) is the local umbrella organization for persons with diverse disabilities. Since 2004, with the aim of improving the health status and quality of life of people with disabilities, the SHDPF has been organizing free annual health examination services for people with disabilities. Those who are voluntarily registered as disabled are eligible to receive health checkups in designated medical institutions every year. Routine health examinations are effective for the early detection and prevention of diseases, which could improve individuals’ quality of life (Qian et al., 2017). However, people with disabilities might undergo screening less frequently due to physical barriers, lack of qualified health care providers, and limited health information for persons with disability (McCarthy, 2014). In light of the fact there are no studies specifically looking at health care access for persons with disabilities in China, a study in United States of America (USA) showed that women with disabilities were significantly less likely to receive pap smears than were women without disabilities (Welner, 1999). This report highlighted the need to collect health information from people with disabilities. Hence, via their free health examination program, the SHDPF has been collecting much-needed surveillance information on the health status of people with diverse disabilities, which provides a unique opportunity to examine health disparities among the education levels in adults with disability. All the health examination records were exported to the Shanghai Disabled Persons’ Rehabilitation Comprehensive Information Platform (SHDPRCIP). This surveillance system was established by the SHDPF to track the health and rehabilitation data of individuals with disabilities in Shanghai. Beyond the health examination records, socio-demographics, disability type and disability severity, which was evaluated by eligible medical doctors based on the International Classification of Functioning, Disability, and Health (ICF), were also collected.

This study aims to examine health disparities in China by comparing health outcomes of adults with disabilities versus those without disabilities and outcomes among disabled adults with different education levels. Our primary hypothesis was that more highly educated adults with disabilities would have better health outcomes. These findings can help assess the significance of these disparities and target interventions toward disabled adults across specific educational levels.

## Materials & Methods

### Data and study design

In this study, we selected all health data from SHDPRCIP on individuals aged 25 or older who received health examinations in 2014. If people underwent two or more examinations, we only used the most recent data. We limited the analyses to participants 25 years of age or older because education level is more stable thereafter (Van der Heide et al., 2013). All records with missing data for the variables of interest were excluded (*n* = 2, 896). The disability types were classified as: visual, hearing, speech, physical, intellectual, mental, and multiple disabilities. However, in this study we combined the hearing and speech disability populations, as in the studies by Zheng et al. (2011).

The annual health checkup included physical examination, diagnostic imaging, and laboratory tests. The physical examination consisted of basic measurements (e.g., height, weight, blood pressure) and organ checkups (e.g., eye, ear, nose, throat, lung, heart, liver, spleen, anorectum). The diagnostic imaging involved chest X-ray, electrocardiogram (ECG), and abdominal ultrasound scan (focusing on the liver, spleen, gallbladder, and pancreas). Finally, the laboratory tests involved routine blood tests, blood biochemical tests, routine urine tests, and immunological tests. During the checkup, a medical practitioner in each department initially gathered preliminary results. Subsequently, all data obtained from the checkup, including the laboratory test results, were integrated and the principal doctor provided the final report to the person with disability.

The institutional review board (IRB) of the Fudan University School of Public Health (IRB #2015-08-0563) approved this study, and prior approval was obtained from the SHDPF about the use of the data in SHDPRCIP.

### Measures

#### Dependent variables

Two diseases (fatty liver and hemorrhoids) and three risk factors (high blood glucose, overweight, and high blood lipids) were selected as dependent variables to reflect the health outcomes of adults with disabilities.

The two diseases were classified using the Tenth Revision of the International Classification of Diseases (ICD-10), with the code K76.0 for fatty liver and I84 for hemorrhoids (Groenen et al., 2007; Peery et al., 2015). Hemorrhoids and fatty liver are two of the highest prevalence diseases in Chinese individuals with disabilities (Kang et al., 2016). Because the obesity and hypertension rates have continued to increase over recent decades, more than 300 million Chinese live with some liver disease, including many disabled Chinese (Wu et al., 2018; Estes et al., 2018). Moreover, hemorrhoids are an increasingly prevalent gastrointestinal disorder in China, contributing to reduced quality of life among those who are disabled (Abramowitz et al., 2019). Understanding the epidemiology of fatty liver and hemorrhoids in individuals with disabilities is important to help guide resource planning for the healthcare system. In addition, we selected 3 key risk factors for chronic conditions: high blood glucose, overweight, and high blood lipids, all of which were frequently observed in disabled Chinese with significant disease burden (Zhang et al., 2018).

Except disease history inquiry, hemorrhoids and fatty liver were diagnosed by the doctors of the rehabilitation centers through the digital rectal exam and abdominal ultrasound scan, respectively. As to overweight, we used the recommended guidelines for the Chinese population of a body mass index (BMI) of ≥24 kg/m^2^ (Zhou, 2002). High blood glucose was defined as a fasting blood glucose of ≥6.1 mmol/L (Chinese Diabetes Federation, 2014). Using the 2016 Chinese Adults’ Prevention and Treatment Guidelines for Dyslipidemia (Hu, 2017), we defined high blood lipids as total cholesterol levels of ≥5.2 mmol/L or triglyceride levels of ≥1.7 mmol/L.

#### Independent variable

The authors defined education level as elementary school education or below, or graduated from middle school, high school, college, or higher. The classification was based on China’s educational system and in accordance with the International Standard Classification of Education (Smyth, 2008; Rong et al., 2017).

### Covariates

Demographic characteristics were regarded as a covariate in this study, including gender (male or female), age (25–29, 30–39, 40–49, 50–59, and 60–69, ≥ 70 years), residence permit (whether they had a rural or urban residence permit), and marital status (never married, married, divorced, or widowed). Disability severity was also included as a covariate in this study, using the four-level classification system in China: level 1 indicates severe disability, level 2 moderately severe disability, level 3 moderate disability, and level 4 mild disability (Bao, 2007).

### Statistical analyses

SPSS Statistics 22.0 was used to analyze all data. The percentage of each education level was calculated for all demographic, disability type, and disability severity groups. Descriptive statistics and Pearson’s chi-squared test were conducted to determine differences in demographic and disability characteristics according to education level. We also compared the distributions of education level between all people without disability in Shanghai, people with disability in Shanghai, and the sample of people with disability in this study to ensure the representativeness of the sample. The data for the first two groups were from the China Statistical Yearbook 2015 (Chinese National Bureau of Statistics, 2015) and the Statistical Bulletin of China Disabled Career Development 2015 (China Disabled Persons’ Federation, 2015). Pearson’s chi-squared test and Fisher’s exact test were used to compare the prevalence of each health outcome across different education levels.

Logistic regression analysis was used to explore differences in health outcomes according to education level after adjusting for covariates. The correlation coefficient of the independent variable and covariates was <0.5, indicating no significant multicollinearity. Individuals with an elementary school education or below were considered as the reference group; odds ratios (ORs) and 95% confidence intervals (CIs) were estimated for the other three education groups. *p*-value <0.05 was regarded as statistically significant.

## Results

In 2014, 46,108 disabled individuals received health examinations. Of these, we selected the 45,611 individuals who were 25 years of age or older. After excluding those with missing data (*n* = 2,896), we were left with an analytical sample of 42,715 individuals with disabilities.

Table 1 presents the descriptive statistics of demographics, disability types, and disability severity. The composition of education levels differed significantly across all demographics, disability types, and disability severity. For example, 52.6% of participants were male. However, among participants in the college or higher education group, 66.5% were male (*p* <0.001). Most participants (72.7%) were aged 50–69 years old, and their average age was 56.7 ±10.5 years. The majority of participants had an urban residence permit (79.1%). However, among participants with an elementary school education or below, only 60.9% had an urban residence permit. Almost all (98.4%) of the disabled adults with a college or higher degree had an urban residence permit. About half of the participants (50.2%) were physically disabled, followed by people with visual disabilities (23.8%). In terms of the severity of the disability, most participants were evaluated as level 4 or level 3, which accounted for 50.4% and 28.8% of the study sample, respectively. As for marital status, the majority of participants (81.6%) were married; only 11.8% of participants were unmarried; and, 6.6% were divorced or widowed.

**Table 1:** Demographics, disability types, and disability severity of disabled adults aged 25 years or older in Shanghai, China.

	**Elementary school or below** (*N* = 10,207) %	**Middle school** (*N* = 21,132) %	**High school** (*N* = 9,509) %	**College or higher** (*N* = 1,867) %	**Total** (*N* = 42,715) %	*p-*value^a^
**Gender**						<0.001
Male	45.9	54.0	53.8	66.5	52.6	
Female	54.1	46.0	46.2	33.5	47.4	
**Age**						<0.001
25–29	3.6	1.5	1.5	3.3	2.1	
30–39	6.9	5.5	4.5	11.2	5.8	
40–49	12.2	12.8	7.4	9.1	11.3	
50–59	24.6	37.8	59.7	22.2	38.9	
60–69	38.2	37.4	20.5	37.0	33.8	
≥ 70	14.4	5.0	6.4	17.2	8.1	
**Residence permit**						<0.001
Rural	39.1	19.6	8.0	1.6	20.9	
Urban	60.9	80.4	92.0	98.4	79.1	
**Disability type**						<0.001
Hearing and speech	10.8	9.8	9.4	11.8	10.0	
Visual	15.2	24.7	29.8	29.5	23.8	
Physical	43.9	52.4	52.1	50.5	50.2	
Intellectual	25.5	7.7	2.2	.4	10.4	
Mental	3.4	4.3	5.5	6.6	4.4	
Multiple	1.2	1.1	1.0	1.2	1.1	
**Disability severity**						<0.001
Level 1	8.7	7.8	7.4	9.1	8.0	
Level 2	10.6	12.8	14.6	16.9	12.8	
Level 3	30.3	29.0	27.2	26.7	28.8	
Level 4	50.4	50.4	50.8	47.3	50.4	
**Marital Status**						<0.001
Never married	16.9	9.4	10.8	16.3	11.8	
Married	76.5	84.3	81.8	77.5	81.6	
Divorced or widowed	6.7	6.3	7.3	6.3	6.6	

**Notes.**

Level 1 indicates the most severe disabilities. Levels 2 and 3 indicate moderately severe to moderate disabilities. Level 4 represents mild disabilities.

a χ2 test was conducted to compare the demographic and disability characteristics of participants by different education levels.

**Figure 1:**
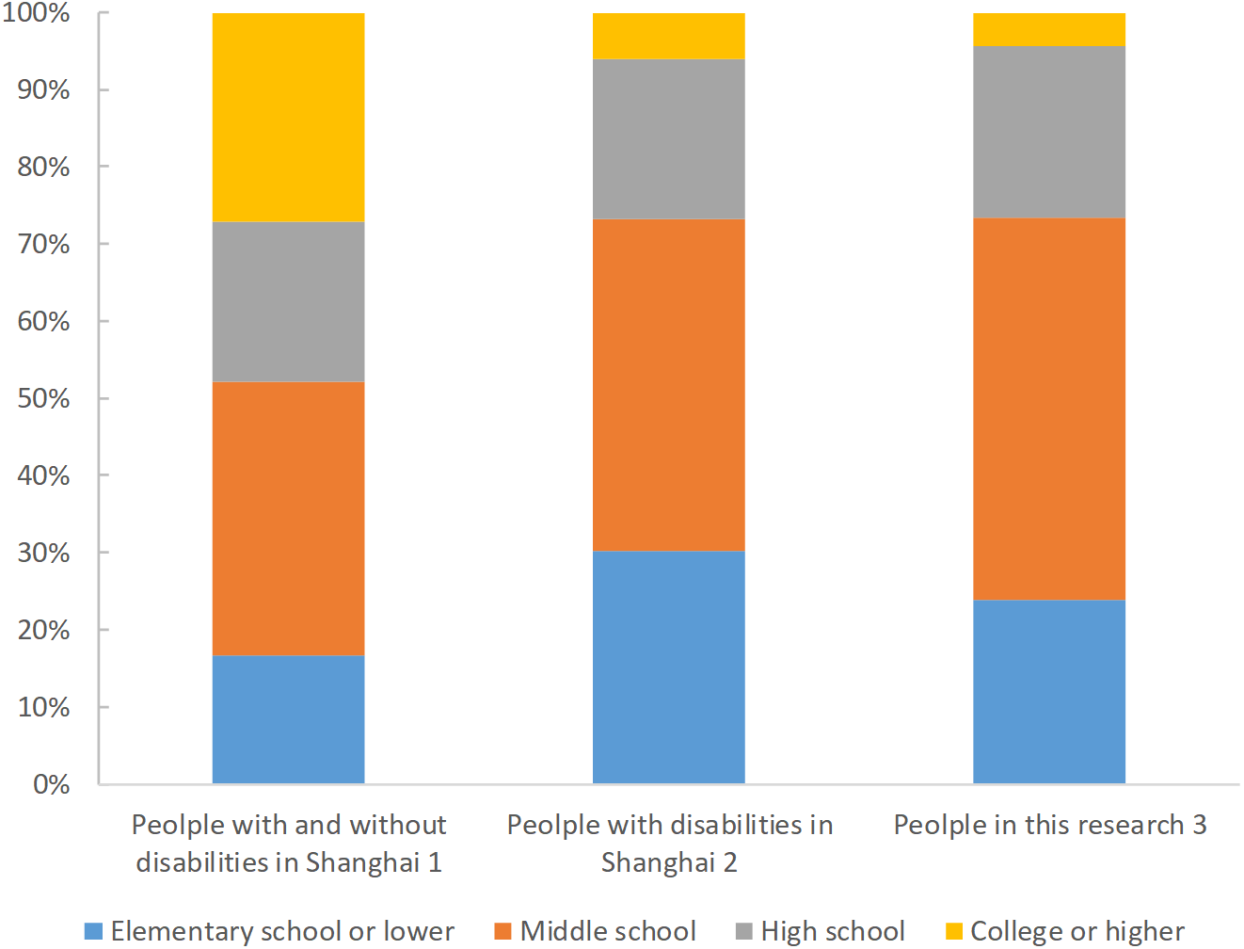
Distributions of education groups in populations with and without disabilities in Shanghai, 2014. Note 1: data from *China Statistical Yearbook 2015*. Note 2: data from *Statistical Bulletin of China Disabled Career Development 2015*. Note 3: data from *The Shanghai Disabled Persons’ Rehabilitation Comprehensive Information Platform*.

Figure 1 shows the distributions of education level among people in Shanghai without disability, people with disability, and the participants in our study. The distributions were similar between people with disabilities in Shanghai and our sample. Of the general population in Shanghai, most had a middle school degree (35.4%), followed by college or higher degree (27.1%), high school degree (20.8%), and elementary school education or below (16.7%). With regard to people with disabilities in Shanghai, the majority of participants (43.0%) had a middle school degree, followed by elementary school education or below (30.2%) and high school degree (20.8%); only 6.0% of participants had a college or higher degree. With respect to our sample, nearly half the participants (49.5%) had a middle school degree and 23.9% had an elementary school education or below. Only 4.4% of participants in this study received a college or higher degree.

Table 2 displays the prevalence of each health outcome according to education level. Nearly half the participants were overweight (BMI ≥ 24; 49.3%), and 58.8% of the participants had high blood lipid levels. The prevalence of fatty liver was 40.6%, with the highest prevalence being found in the college education or higher group (42.5%). People with an elementary school education or below had the highest prevalence of overweight (52.1%) and high blood glucose (20.8%), but the lowest prevalence of hemorrhoids (18.6%) and fatty liver (38.9%). Individuals with a high school education had the highest prevalence of hemorrhoids (30.5%).

**Table 2:** Health conditions among each education level across disability types and disability severity.

**Disability type/ disability severity**	**Education level**	**Overweight (BMI ≥ 24)**	**Hemorrhoids**	**Fatty liver**	**High blood glucose**	**High blood lipid**
		*n* (%)	*n* (%)	*n* (%)	*n* (%)	*n* (%)
**Total**	Elementary school or below	5,317 (52.1)	1,898 (18.6)	3,972 (38.9)	2,120 (20.8)	5,833 (57.1)
	Middle school	10,359 (49.0)	5,753 (27.2)	8,609 (40.7)	4,244 (20.1)	12,382 (58.6)
	High school	4,487 (47.2)	2,904 (30.5)	3,977 (41.8)	1,879 (19.8)	5,835 (61.4)
	College or higher	905 (48.5)	565 (30.3)	794 (42.5)	304 (16.3)	1,072 (57.4)
	Total	21,068 (49.3)	11,120 (26.0)	17,352 (40.6)	8,547 (20.0)	25,122 (58.8)
	*p*-value^a^	<0 .001	<0.001	<0 .001	<0 .001	<0.001
**Hearing and speech**	Elementary school or below	547 (49.7)	269 (24.4)	402 (36.5)	229 (20.8)	635 (57.7)
	Middle school	912 (44.1)	638 (30.9)	836 (40.4)	417 (20.2)	1,184 (57.3)
	High school	373 (41.7)	313 (35.0)	357 (39.9)	150 (16.8)	527 (58.9)
	College or higher	92 (41.6)	73 (33.0)	92 (41.6)	35 (15.8)	125 (56.6)
	Subtotal	1,924 (44.9)	1,293 (30.2)	1,687 (39.4)	831 (19.4)	2,471 (57.7)
	*p*-value^a^	0.001	<0.001	0.151	0 .047	0.850
**Visual**	Elementary school or below	845 (54.6)	394 (25.5)	599 (38.7)	380 (24.5)	919 (59.4)
	Middle school	2,395 (45.8)	1,820 (34.8)	2,227 (42.6)	1,145 (21.9)	3,227 (61.7)
	High school	1,253 (44.2)	978 (34.5)	1,221 (43.1)	589 (20.8)	1,801 (63.6)
	College or higher	253 (46.0)	175 (31.8)	243 (44.2)	100 (18.2)	314 (57.1)
	Subtotal	4,746 (46.7)	3,367 (33.1)	4,290 (42.2)	2,214 (21.8)	6,261 (61.6)
	*p*-value^a^	<0.001	<0.001	0.019	0.005	0.005
**Physical**	Elementary school or below	2,384 (53.2)	772 (17.2)	1,778 (39.7)	928 (20.7)	2,719 (60.7)
	Middle school	5,619 (50.7)	2,771 (25.0)	4,426 (40.0)	2,194 (19.8)	6,542 (59.1)
	High school	2,407 (48.6)	1,424 (28.7)	2,044 (41.2)	978 (19.7)	3,040 (61.3)
	College or higher	467 (49.5)	289 (30.6)	395 (41.9)	149 (15.8)	540 (57.3)
	Subtotal	10,877 (50.7)	5,256 (24.5)	8,643 (40.3)	4,249 (19.8)	12,841 (59.9)
	*p*-value^a^	<0.001	<0.001	0.258	0.008	0.010
**Intellectual**	Elementary school or below	1,277 (49.1)	401 (15.4)	1,018 (39.1)	472 (18.1)	1,262 (48.5)
	Middle school	796 (48.9)	263 (16.2)	676 (41.5)	239 (14.7)	774 (47.6)
	High school	107 (51.4)	31 (14.9)	91 (43.8)	35 (16.8)	99 (47.6)
	College or higher	3 (42.9)	2 (28.6)	2 (28.6)	1 (14.3)	2 (28.6)
	Subtotal	2,183 (49.1)	697 (15.7)	1,787 (40.2)	747 (16.8)	2,137 (48.1)
	*p*-value^a^	0.900	0.704	0.265	0.036	0.696
**Mental**	Elementary school or below	207 (59.0)	39 (11.1)	119 (33.9)	93 (26.5)	230 (65.5)
	Middle school	533 (59.3)	188 (20.9)	353 (39.3)	201 (22.4)	510 (56.7)
	High school	305 (58.1)	130 (24.8)	224 (42.7)	111 (21.1)	313 (59.6)
	College or higher	84 (67.7)	23 (18.5)	51 (41.1)	16 (12.9)	76 (61.3)
	Subtotal	1,129 (59.5)	380 (20.0)	747 (39.3)	421 (22.2)	1,129 (59.5)
	*p*-value^a^	0.264	<0.001	0.073	0.016	0.040
**Multiple**	Elementary school or below	57 (45.6)	23 (18.4)	56 (44.8)	18 (14.4)	68 (54.4)
	Middle school	104 (44.4)	73 (31.2)	91 (38.9)	48 (20.5)	145 (62.0)
	High school	42 (45.2)	28 (30.1)	40 (43.0)	16 (17.2)	55 (59.1)
	College or higher	6 (27.3)	3 (13.6)	11 (50.0)	3 (13.6)	15 (68.2)
	Subtotal	209 (44.1)	127 (26.8)	198 (41.8)	85 (17.9)	283 (59.7)
	*p*-value^a^	0.441	0.026	0.585	0.488	0.452
**Level 1**	Elementary school or below	451 (51.0)	198 (22.4)	354 (40.0)	168 (19.0)	521 (58.9)
	Middle school	787 (47.8)	458 (27.8)	688 (41.8)	356 (21.6)	1,001 (60.9)
	High school	322 (45.5)	197 (27.8)	300 (42.4)	137 (19.4)	428 (60.5)
	College or higher	63 (37.1)	41 (24.1)	78 (45.9)	25 (14.7)	91 (53.5)
	Subtotal	1,623 (47.6)	894 (26.2)	1,420 (41.7)	686 (20.1)	2,041 (59.9)
	*p*-value^a^	0.005	0.016	0.491	0.092	0.265
**Level 2**	Elementary school or below	547 (50.3)	177 (16.3)	404 (37.2)	215 (19.8)	586 (53.9)
	Middle school	1,372 (50.9)	673 (25.0)	1,057 (39.2)	615 (22.8)	1,554 (57.7)
	High school	668 (48.2)	394 (28.4)	572 (41.3)	277 (20.0)	858 (61.9)
	College or higher	170 (53.8)	75 (23.7)	133 (42.1)	49 (15.5)	178 (56.3)
	Subtotal	2,757 (50.3)	1,319 (24.1)	2,166 (39.5)	1,156 (21.1)	3,176 (57.9)
	*p*-value^a^	0.222	<0.001	0.153	0.005	0.001
**Level 3**	Elementary school or below	1,620 (52.4)	491 (15.9)	1,184 (38.3)	602 (19.5)	1,787 (57.8)
	Middle school	3,006 (49.0)	1,444 (23.5)	2,482 (40.4)	1,063 (17.3)	3,523 (57.4)
	High school	1,207 (46.7)	704 (27.2)	1,071 (41.4)	496 (19.2)	1,534 (59.3)
	College or higher	237 (47.6)	134 (26.9)	214 (43.0)	76 (15.3)	294 (59.0)
	Subtotal	6,070 (49.3)	2,773 (22.5)	4,951 (40.2)	2,237 (18.2)	7,138 (58.0)
	*p*-value^a^	<0.001	<0.001	0.046	0.011	0.387
**Level 4**	Elementary school or below	2,699 (52.5)	1,032 (20.1)	2,030 (39.5)	1,135 (22.1)	2,939 (57.1)
	Middle school	5,194 (48.8)	3,178 (29.8)	4,382 (41.1)	2,210 (20.7)	6,304 (59.2)
	High school	2,290 (47.4)	1,609 (33.3)	2,034 (42.1)	969 (20.1)	3,015 (62.4)
	College or higher	435 (49.3)	315 (35.7)	369 (41.8)	154 (17.4)	509 (57.6)
	Subtotal	10,618 (49.4)	6,134 (28.5)	8,815 (41.0)	4,468 (20.8)	12,767 (59.4)
	*p*-value^a^	<0.001	<0.001	0.051	0.005	<0.001

**Notes.**

a χ2 test and Fisher’s exact test were conducted to compare the prevalence of each health outcome in different education levels.

As for disability type, people with visual disability had the highest prevalence of hemorrhoids (33.1%), fatty liver (42.2%), and high blood lipids (61.6%). Participants with mental disability had the highest prevalence of overweight (59.5%) and high blood glucose (22.2%), but the lowest prevalence of fatty liver (39.3%). Regarding disability severity, participants with severe disability (level 1) had the highest prevalence of fatty liver (41.7%) and high blood lipids (59.9%). People with moderately severe disability (level 2) had the highest prevalence of overweight (50.3%) and high blood glucose (21.1%), but the lowest prevalence of fatty liver (39.5%) and high blood lipids (57.9%). People with mild disability (level 4) had the highest prevalence of hemorrhoids (28.5%).

**Figure 2:**
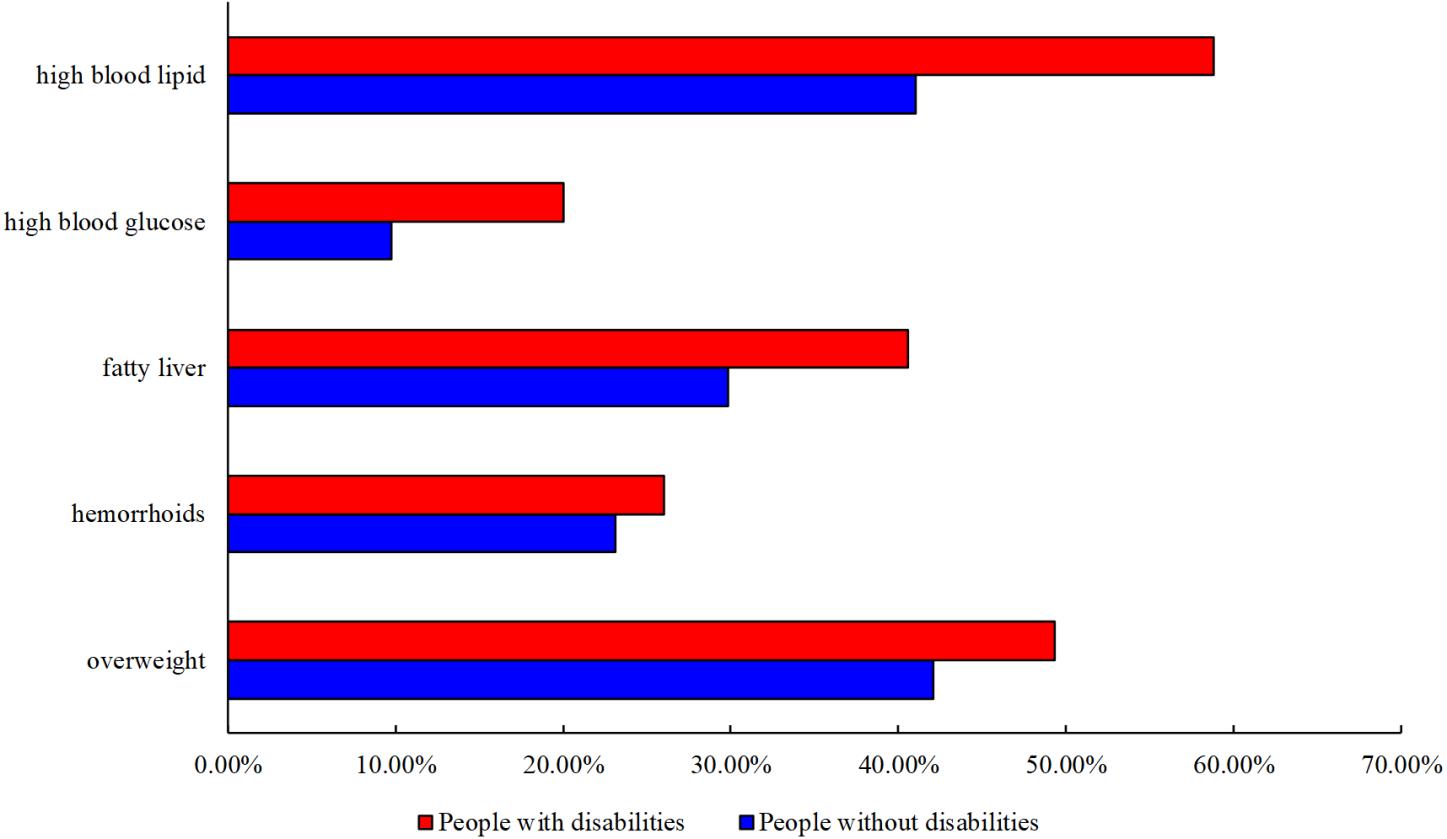
Comparison of Prevalence of Each Health Outcome between People with and without Disabilities.

Figure 2 shows the comparison of prevalence of each health outcome between people with and without disabilities. Compared with their peers without disabilities, the prevalence of all the studied health outcomes was higher among people with disabilities: overweight (without disabilities versus with disabilities), 42.1% versus 49.3%; hemorrhoids, 23.1% versus 26.0%; fatty liver, 29.8% versus 40.6%; high blood glucose, 9.7% versus 20.0% and high blood lipid, 41.0% versus 58.8% (Li & Wang, 2017; Zhao, Zhai & Hu, 2006; Zeng et al., 2016; Wang, Zhang & Guo, 2011; Zang et al., 2012; Zhou, 2014; Shang, Chen & Li, 2015; Han & Gao, 2013; Xiao, Peng & Ren, 2011; Zhang et al., 2012; Sun, 2010; Jia, Wang & Pan, 2006; Xu et al., 2014).

Table 3 presents the results of the regression analysis. After adjusting for all covariates, we found that the relation between education level and health differed according to the specific disease or risk factors. As to adults with speech and hearing, visual, and physical disabilities, those with a middle school education had significantly lower odds of being overweight than did those with an elementary school education or below (hearing/speaking aOR = 0.81, *p* < .01; visual aOR = 0.71, *p* < .001; physical aOR = 0.90, *p* < .01). Among people with visual and physical disabilities, those with a college education or higher had significantly lower odds of having high blood glucose (visual aOR = 0.68, *p* < .01; physical aOR = 0.73, *p* < .01) than did those with an elementary school education or below.

In contrast, compared with the other educational levels, people with an elementary school education or below had significantly lower odds of hemorrhoids for hearing/speaking, visual, physical, and mental disabilities. Among people with visual disabilities, the odds of having fatty liver rose with education level (middle school aOR = 1.19, *p* < .001; high school aOR = 1.22, *p* < .001; college or higher aOR = 1.27, *p* < .05). Interestingly, education level was not significantly related to any health outcomes in adults with intellectual or multiple disabilities.

## Discussion

Drawing on a unique dataset containing rich information, we provide detailed evidence of the association between education and health outcomes among adults with disability living in Shanghai, China, and compare health outcomes of adults with disabilities to those without disabilities.

We found that the relationship of education level with health outcomes differed according to the health condition and disability type. As hypothesized, overweight and high blood glucose were more prevalent in lower educational groups of people with disability, which is in accordance with findings in people without disability (Hu et al., 2016). Different mechanisms for the positive influence of education level on health have been proposed. For example, education might defend against disease by cultivating a more positive lifestyle, problem-solving abilities, and aspiring values (Winkleby, Fortmann & Barrett, 1990). In addition, education helps to cultivate positive social and psychological abilities, and it insulates individuals against disadvantageous influences (Williams, 1990).

**Table 3:** Results of logistic regressions of health outcomes with education level across disability types.

	**BMI ≥ 24**				**Haemorrhoid**				**Fatty liver**				**High blood glucose**				**High blood lipid**			
	**OR**	**95% CI**		*p*	**OR**	**95% CI**		*p*	**OR**	**95% CI**		*p*	**OR**	**95% CI**		*p*	**OR**	**95% CI**		**p**
**Total**																				
**Education**^a^																				
Elementary school or below	Reference				Reference				Reference				Reference				Reference			
Middle school	0.85	0.81	0.90	<0 .001	1.76	1.65	1.88	<0 .001	1.08	1.02	1.14	0.005	0.95	0.89	1.01	0.087	1.01	0.96	1.07	0.655
High school	0.80	0.75	0.85	<0 .001	2.03	1.88	2.19	<0 .001	1.12	1.05	1.20	<0 .001	0.91	0.84	0.98	0.015	1.08	1.01	1.15	0.018
College or higher	0.80	0.72	0.89	<0 .001	2.21	1.96	2.49	<0 .001	1.15	1.03	1.27	0.009	0.70	0.61	0.80	<0 .001	1.06	0.96	1.18	0.268
**Hearing & Speech**																				
**Education**^b^																	
Elementary school or below	Reference				Reference				Reference				Reference				Reference			
Middle school	0.81	0.69	0.94	0.007	1.60	1.33	1.92	<0 .001	1.14	0.97	1.34	0.103	1.02	0.84	1.24	0.840	1.01	0.86	1.18	0.951
High school	0.73	0.60	0.89	0.002	1.89	1.51	2.36	<0 .001	1.10	0.90	1.34	0.351	0.77	0.60	0.99	0.043	1.07	0.88	1.31	0.485
College or higher	0.69	0.51	0.95	0.021	2.20	1.56	3.10	<0 .001	1.24	0.91	1.69	0.169	0.78	0.52	1.17	0.229	1.30	0.95	1.78	0.106
**Visual Disability**																				
**Education**^b^																				
Elementary school or below	Reference				Reference				Reference				Reference				Reference			
Middle school	0.71	0.63	0.80	<0 .001	1.79	1.55	2.06	<0 .001	1.19	1.05	1.34	0.007	0.91	0.79	1.05	0.192	1.10	0.97	1.25	0.129
High school	0.69	0.60	0.79	<0 .001	1.82	1.56	2.13	<0 .001	1.22	1.06	1.40	0.005	0.88	0.74	1.03	0.115	1.16	1.01	1.34	0.040
College or higher	0.67	0.55	0.82	<0 .001	1.92	1.52	2.41	<0 .001	1.27	1.03	1.55	0.024	0.68	0.53	0.88	0.003	1.06	0.86	1.31	0.561
**Physical Disability**																				
**Education**^b^																				
Elementary school or below	Reference				Reference				Reference				Reference				Reference			
Middle school	0.90	0.83	0.96	0.004	1.99	1.81	2.19	<0 .001	1.01	0.93	1.09	0.866	1.00	0.91	1.10	0.989	1.01	0.94	1.09	0.798
High school	0.84	0.77	0.91	<0 .001	2.46	2.20	2.75	<0 .001	1.05	0.96	1.15	0.257	0.99	0.89	1.11	0.935	1.09	1.00	1.20	0.059
College or higher	0.86	0.74	0.99	0.041	2.84	2.40	3.36	<0 .001	1.06	0.91	1.23	0.448	0.73	0.60	0.89	0.002	1.07	0.92	1.24	0.407
**Intellectual Disability**																				
**Education**^b^																				
Elementary school or below	Reference				Reference				Reference				Reference				Reference			
Middle school	0.97	0.85	1.10	0.606	1.11	0.93	1.33	0.248	1.13	0.99	1.29	0.063	0.77	0.65	0.92	0.004	1.03	0.90	1.17	0.694
High school	1.07	0.80	1.43	0.635	0.93	0.61	1.40	0.716	1.24	0.93	1.66	0.148	0.83	0.56	1.22	0.335	0.96	0.72	1.29	0.803
College or higher	0.75	0.17	3.36	0.703	2.90	0.52	16.22	0.225	0.66	0.13	3.43	0.623	1.01	0.12	8.57	0.996	0.59	0.11	3.10	0.535
**Mental Disability**																				
**Education**^b^																			
Elementary school or below	Reference				Reference				Reference				Reference				Reference			
Middle school	0.96	0.73	1.25	0.736	2.31	1.55	3.43	<0 .001	1.26	0.96	1.66	0.097	0.91	0.67	1.24	0.546	0.72	0.55	0.95	0.020
High school	0.88	0.65	1.20	0.412	3.01	1.94	4.68	<0 .001	1.43	1.05	1.96	0.025	0.88	0.61	1.26	0.486	0.84	0.62	1.15	0.290
College or higher	1.23	0.77	1.96	0.381	2.35	1.27	4.35	0.007	1.38	0.88	2.17	0.165	0.54	0.29	1.01	0.053	0.99	0.63	1.56	0.971
**Multiple Disabilities**																				
**Education**^b^																				
Elementary school or below	Reference				Reference				Reference				Reference				Reference			
Middle school	0.92	0.57	1.50	0.739	1.62	0.88	2.99	0.123	0.83	0.51	1.35	0.454	1.74	0.88	3.44	0.111	1.32	0.80	2.18	0.274
High school	0.88	0.48	1.63	0.693	1.58	0.75	3.34	0.229	1.07	0.57	1.98	0.840	1.22	0.53	2.82	0.643	1.09	0.58	2.06	0.781
College or higher	0.43	0.16	1.21	0.112	0.63	0.16	2.45	0.509	1.49	0.58	3.82	0.411	0.84	0.21	3.28	0.801	1.58	0.57	4.33	0.377

**Notes.**

a Adjusted for gender, age, residence permit, marital status, disability type, and disability severity.

b Adjusted for gender, age, residence permit, marital status and disability severity.

OR odds ratio CI confidence interval

However, contrary to our hypothesis, education was not always associated with better health in adults with disabilities. For example, the prevalence of hemorrhoids, fatty liver and high blood lipids were higher among individuals with disabilities who had a higher education level. This is in line with the findings of Leeves & Soyiri (2015). One possible explanation for this finding is that more educated individuals have greater psychological needs and more life pursuits. To achieve these pursuits, they must devote greater time and energy, even at the expense of their health. Second, individuals with a higher education level tend to utilize computers and other electronic equipment more frequently and for longer periods of time, which results in less physical activity and a higher risk of vision loss, spinal disease, anxiety, and depression (Marmot et al., 1998; Li & Wang, 2017). In addition, in comparison with less-educated people, who primarily engage in manual labor, more-educated people might require greater strength and resilience to achieve greater job expertise. Currently, the government provides some protection for the personal safety and economic security of manual laborers. However, less attention and compensation is given to addressing the mental stress often experienced by more-educated people (Zhao & Hu, 2016). Moreover, in developing countries, people with higher education levels tend to have higher incomes, making it easier for them to access unhealthy foods, tobacco, or alcohol, which are key risk factors for both hemorrhoids and fatty liver (Zhong, 2015).

For people with intellectual disabilities, the association between education and health outcomes was not significant. This finding differs from that of Landes (Landes, 2017), who found that education was a predictor of health outcomes for adults with intellectual disability in the USA, and that increased education was associated with a lower mortality risk among adults with intellectual disability (Landes, 2017). A major reason for the different findings is that the education system for people with intellectual disabilities differs extensively between China and the USA. In US, improvement in educational opportunities for people with intellectual disability began in the 1940s and has since increased the access of people with such disabilities to the public education system and higher-quality educational programs (Landes, 2017). In China, educational support still cannot meet the demands of people with intellectual disability (Wu et al., 2010). Currently, there are three main modes of education for people with intellectual disability in China: isolated special-education schools and classes, special classes at ordinary schools, and ordinary classes at ordinary schools. Data from the Second China National Disability Sample Survey revealed that only 5.5% of people with intellectual disability received a general education at ordinary schools, 1.5% were enrolled in special education classes at ordinary schools, and 3.4% were being educated in isolated special schools (Wu et al., 2010). In addition, we chose specific health outcomes, whereas Landes focused on mortality (Landes, 2017). We also selected different control variables, and included disability severity, which was not considered in Landes (Landes, 2017). Finally, there are significant differences in health behaviors and health care services between the USA and China, particularly in relation to eating habits, sports activities, social inclusion, and health policy, all of which could have influenced the association between education and health outcomes in adults with disability as well (Eide & Showalter, 2011).

Although the huge direct burden of disability has been made clear in previous studies (Loyalka et al., 2014), the physical health status of individuals with disabilities also requires attention. Our results found that adults with disabilities in Shanghai had worse health outcomes than the general population without disabilities, which is in line with past findings (Xu, 2014). Compared with adults without disabilities, adults with disabilities have restrictions on their daily activities and physical exercise that are imposed by these disabilities, which would influence their metabolism. The Shanghai government has invested resources in physical activities facilities for the general population, but these are not necessarily accessible by the disabled (Chen et al., 2018; Liu et al., 2017). More equipment or venues can be added to accommodate disabled individuals in Shanghai. In addition, people with disabilities have greater unmet health and rehabilitation needs than their peers without disabilities and face more obstacles in accessing mainstream health care services (Tomlinson et al., 2009).

Our results revealed that individuals with disabilities have less access to higher education. The first reason for this finding might be the traditional bias in China, which holds that admitting people with disabilities to college is a waste of resources, since they do not have the ability to accept postsecondary education (Wang, 2009). Second, there are obvious limitations in the current higher education system for people with disabilities in China (Liu, Wu & Zou, 2016). These include the small scale of the career paths and limited majors available and the lack of testing accommodations (Deng, Poon-Mcbrayer & Farnsworth, 2001; Tian & Wei, 2015). Education is an important path for maximizing the developmental potential of each individual with disability. It can help empower their family and promote their contribution to society. To improve education among the disabled population, a first step might be helping parents, teachers, social workers, and the general public to understand the significance of proper nutrition, medical care, education, and safety for the optimal development of people with disabilities (Chen & Simeonsson, 1993). Additionally, the systematic cultivation of special educators who can take on leadership roles in higher education institutions must be established. Moreover, enrollment rates in special education courses in normal universities should be increased, and relevant courses on special needs should be provided to students willing to work with people with disabilities (The State Council of the People’s Republic of China, 2014). To achieve these improvements, greater importance should be attached to special and inclusive education. For example, in 2014 the China National Special Education Promotion Plan was announced, which was intended to boost inclusive education, particularly by enhancing accessibility to it, strengthening education conditions, and raising the teaching and learning quality of children with special needs (The State Council of the People’s Republic of China, 2014).

Taken together, our findings indicate an urgent need to promote both the education and health of people with disabilities in China. Promoting the health of people with disabilities is a Healthy China 2030 goal (The Lancet, 2016). Empirical research is required to explore the best educational approaches for people with disabilities, many of whom might not utilize traditional strategies such as radio public service announcements or television and printed materials. Teachers in the special education system and disability service providers should partner with health educators to make sure that they all have the essential information, support, and training to provide accessible services to individuals with disabilities.

## Limitations and Implications

This study has a few limitations. First, the cross-sectional data do not allow for causal inferences about the findings. In addition, although we identified a strong association between education level and different aspects of health outcomes among people with disabilities, the specific mechanisms of this association have not been explored extensively. Moreover, this was a registry-based study; some disabled people in China choose to remain unregistered, which might create a selection bias for certain groups with severe disabilities. However, our study still represents the disabled population in Shanghai because of the large sample size and real-world setting. Another limitation is that we likely have omitted some important related variables (e.g., social support, living environment, medical care accessibility) and information (e.g., medical devices). Despite these limitations, this is one of the first studies in China to examine education as a determinant of health among individuals with disabilities. While acknowledging the complex relation between education and health, future research should leverage our study to design effective interventions and prevention methods to improve the health outcomes of people with disability in China and other developing countries.

## Conclusion

There may be a complex relationship between education and health, especially among adults with disabilities in China. The association between education and health outcomes differed according to the health condition and disability type. Compared with people without disabilities, adults with disabilities in Shanghai have relatively poor health. To reduce the prevalence rate of overweight and high blood glucose among people with disabilities, tailored health promotion initiatives must be developed for people with lower education levels. In contrast, specific attention should be paid to the prevention of hemorrhoids and fatty liver among more-educated people with disabilities. Recognition of the complexity of this association should be embedded in future prospective research and intervention programs to address health and education disparities in people with disabilities in China.

##  Supplemental Information

10.7717/peerj.6382/supp-1 Data S1 Raw data

10.7717/peerj.6382/supp-2 Supplemental Information 1 Values and categories of the variables in raw data

10.7717/peerj.6382/supp-3 Table S1 Table 1 with exact *p*-value Note: Level 1 indicates the most severe disabilities. Levels 2 and 3 indicate moderately severe to moderate disabilities. Level 4 represents mild disabilities. ∗*χ*2 test was conducted to compare the demographic and disability characteristics of participants by different education levels. a, *p* = 1.14896317009354E–75 b, *p* = 0.000 c, *p* = 0.000 d, *p* = 0.000 e, *p* = 5.40799275230419E–22 f, *p* = 1.09432772270004E–90.

10.7717/peerj.6382/supp-4 Table S2 Table 2 with exact *p*-value ∗*χ*2 test and Fisher’s exact test were conducted to compare the prevalence of each health outcome in different education levels. a, *p* = 8.08620222642061E–11 b, *p* = 4.12037312310817E–92 c, *p* = 0.000107210522360043 d, *p* = 0.000144998989757823 e, *p* = 1.62261094666859E–08 f, *p* = 3.12302572411109E–06 g, *p* = 2.94192672999227E–10 h, *p* = 5.51669620111951E–11 i, *p* = 0.000111660991424272 j, *p* = 2.04694838804187E–42 k, *p* = 0.0000127694131019277 l, *p* = 3.46362411394606E–11 m, *p* = 0.000185619627816675 n, *p* = 7.79213264752363E–26 o, *p* = 2.40217947128789E–06 p, *p* = 2.26450131270884E–57 q, *p* = 1.02180388285092E–06.

10.7717/peerj.6382/supp-5 Table S3 Table 3 with exact p-value ^1^Adjusted for gender, age, *hukou*, marital status, disability type, and disability severity. ^2^Adjusted for gender, age, *hukou*, marital status and disability severity. OR, odds ratio; CI, confidence interval a, *p* = 1.82610338670946E–09 b, *p* = 3.59421532803415E–65 c, *p* = 5.21541397814597E–12 d, *p* = 5.34560735401499E–73 e, *p* = 0.000485652226983674 f, *p* = 0.0000228939475230585 g, *p* = 1.46498546264525E–38 h, *p* = 3.36865066099832E–07 i, *p* = 5.65373183403489E–07 j, *p* = 2.05001733219406E–08 k, *p* = 0.0000069875277559385 l, *p* = 2.0130374120598E–08 m, *p* = 5.73649683295901E–16 n, *p* = 9.50661598792444E–08 o, *p* = 9.00383872834067E–14 p, *p* = 0.000111084046409749 q, *p* = 3.22739499284614E–08 r, *p* = 1.23882486591996E–44 s, *p* = 0.0000848185284451649 t, *p* = 2.57456171857619E–56 u, *p* = 2.12628072004128E–33 v, *p* = 0.0000379636759741926 w, *p* = 9.13903589573368E–07.

## Additional Information and Declarations

### Data Availability

The following information was supplied regarding data availability: The raw data is available in the Supplemental Files.
