# Supplementary material for: Association between education and health outcomes among adults with disabilities: evidence from Shanghai, China
Source: PeerJ. 2019 Feb 19;7:e6382. doi: 10.7717/peerj.6382 (PMC6385680; doi:10.7717/peerj.6382)
Supplement: Supplemental Information 1 [file peerj-07-6382-s002.docx]

| **Values** | **Covariates** | | | | | | **Independent Variable** | **Dependent variables** | | | | |
| --- | --- | --- | --- | --- | --- | --- | --- | --- | --- | --- | --- | --- |
|  | Gender | Age | Residence Permit | Marital Status | Disability Type | Disability Severity | Education Level | Overweight | Hemorrhoids | Fatty Liver | High Blood Glucose | High Blood Lipid |
| 0 |  |  |  |  |  |  |  | No | No | No | No | No |
| 1 | Male | 25-26 | Rural | Never married | Hearing and speech | Level 1 | Elementary school or below | Yes | Yes | Yes | Yes | Yes |
| 2 | Female | 30-39 | Urban | Married | Visual | Level 2 | Middle school |  |  |  |  |  |
| 3 |  | 40-49 |  | Divorced or widowed | Physical | Level 3 | High school |  |  |  |  |  |
| 4 |  | 50-59 |  |  | Intellectual | Level 4 | College or higher |  |  |  |  |  |
| 5 |  | 60-69 |  |  | Mental |  |  |  |  |  |  |  |
| 6 |  | >=70 |  |  | Multiple |  |  |  |  |  |  |  |

**Table. Values /categories of the variables**
