# Supplementary material for: Association between education and health outcomes among adults with disabilities: evidence from Shanghai, China
Source: PeerJ. 2019 Feb 19;7:e6382. doi: 10.7717/peerj.6382 (PMC6385680; doi:10.7717/peerj.6382)
Supplement: Table S1 — Note: Level 1 indicates the most severe disabilities. Levels 2 and 3 indicate moderately severe to moderate disabilities. Level 4 represents mild disabilities. ∗χ2 test was conducted to compare the demographic and disability characteristics of participants by different education levels. a, p = 1.14896317009354E–75 b, p = 0.000 c, p = 0.000 d, p = 0.000 e, p = 5.40799275230419E–22 f, p = 1.09432772270004E–90. [file peerj-07-6382-s003.docx]

**Table 1. Demographics, Disability Types, and Disability Severity of Disabled Adults Aged 25 Years or Older in Shanghai, China**

|  | **Elementary school or below** | **Middle school** | **High school** | **College or higher** | **Total** |  |
| --- | --- | --- | --- | --- | --- | --- |
|  | **(N=10207)** | **(N=21132)** | **(N=9509)** | **(N=1867)** | **(N=42715)** |  |
|  | **%** | **%** | **%** | **%** | **%** | ***p-*value*** |
| **Gender** |  |  |  |  |  | <0.001^a^ |
| Male | 45.9 | 54.0 | 53.8 | 66.5 | 52.6 |  |
| Female | 54.1 | 46.0 | 46.2 | 33.5 | 47.4 |  |
| **Age** |  |  |  |  |  | <0.001^b^ |
| 25-29 | 3.6 | 1.5 | 1.5 | 3.3 | 2.1 |  |
| 30-39 | 6.9 | 5.5 | 4.5 | 11.2 | 5.8 |  |
| 40-49 | 12.2 | 12.8 | 7.4 | 9.1 | 11.3 |  |
| 50-59 | 24.6 | 37.8 | 59.7 | 22.2 | 38.9 |  |
| 60-69 | 38.2 | 37.4 | 20.5 | 37.0 | 33.8 |  |
| ≥70 | 14.4 | 5.0 | 6.4 | 17.2 | 8.1 |  |
| **Residence permit** |  |  |  |  |  | <0.001^c^ |
| Rural | 39.1 | 19.6 | 8.0 | 1.6 | 20.9 |  |
| Urban | 60.9 | 80.4 | 92.0 | 98.4 | 79.1 |  |
| **Disability type** |  |  |  |  |  | <0.001^d^ |
| Hearing and speech | 10.8 | 9.8 | 9.4 | 11.8 | 10.0 |  |
| Visual | 15.2 | 24.7 | 29.8 | 29.5 | 23.8 |  |
| Physical | 43.9 | 52.4 | 52.1 | 50.5 | 50.2 |  |
| Intellectual | 25.5 | 7.7 | 2.2 | .4 | 10.4 |  |
| Mental | 3.4 | 4.3 | 5.5 | 6.6 | 4.4 |  |
| Multiple | 1.2 | 1.1 | 1.0 | 1.2 | 1.1 |  |
| **Disability severity** |  |  |  |  |  | <0.001^e^ |
| Level 1 | 8.7 | 7.8 | 7.4 | 9.1 | 8.0 |  |
| Level 2 | 10.6 | 12.8 | 14.6 | 16.9 | 12.8 |  |
| Level 3 | 30.3 | 29.0 | 27.2 | 26.7 | 28.8 |  |
| Level 4 | 50.4 | 50.4 | 50.8 | 47.3 | 50.4 |  |
| **Marital Status** |  |  |  |  |  | <0.001^f^ |
| Never married | 16.9 | 9.4 | 10.8 | 16.3 | 11.8 |  |
| Married | 76.5 | 84.3 | 81.8 | 77.5 | 81.6 |  |
| Divorced or widowed | 6.7 | 6.3 | 7.3 | 6.3 | 6.6 |  |

Note: Level 1 indicates the most severe disabilities. Levels 2 and 3 indicate moderately severe to moderate disabilities. Level 4 represents mild disabilities.

*χ2 test was conducted to compare the demographic and disability characteristics of participants by different education levels.

^a^ p = 1.14896317009354E-75 ^b^ p = 0.000 ^c^ p = 0.000 ^d^ p = 0.000 ^e^ p = 5.40799275230419E-22 ^f^ p = 1.09432772270004E-90
