# Supplementary material for: Association between education and health outcomes among adults with disabilities: evidence from Shanghai, China
Source: PeerJ. 2019 Feb 19;7:e6382. doi: 10.7717/peerj.6382 (PMC6385680; doi:10.7717/peerj.6382)
Supplement: Table S2 — ∗χ2 test and Fisher’s exact test were conducted to compare the prevalence of each health outcome in different education levels. a, p = 8.08620222642061E–11 b, p = 4.12037312310817E–92 c, p = 0.000107210522360043 d, p = 0.000144998989757823 e, p = 1.62261094666859E–08 f, p = 3.12302572411109E–06 g, p = 2.94192672999227E–10 h, p = 5.51669620111951E–11 i, p = 0.000111660991424272 j, p = 2.04694838804187E–42 k, p = 0.0000127694131019277 l, p = 3.46362411394606E–11 m, p = 0.000185619627816675 n, p = 7.79213264752363E–26 o, p = 2.40217947128789E–06 p, p = 2.26450131270884E–57 q, p = 1.02180388285092E–06. [file peerj-07-6382-s004.docx]

**Table 2. Health Conditions among Each Education Level across Disability Types and Disability Severity.**

| **Disability Type/ Disability Severity** | **Education Level** | **Overweight (BMI≥24)** | **Hemorrhoids** | **Fatty Liver** | **High Blood Glucose** | **High Blood Lipid** |
| --- | --- | --- | --- | --- | --- | --- |
|  |  | n (%) | n (%) | n (%) | n (%) | n (%) |
| **Total** | **Elementary school or below** | 5317(52.1) | 1898(18.6) | 3972(38.9) | 2120(20.8) | 5833(57.1) |
|  | **Middle school** | 10359(49.0) | 5753(27.2) | 8609(40.7) | 4244(20.1) | 12382(58.6) |
|  | **High school** | 4487(47.2) | 2904(30.5) | 3977(41.8) | 1879(19.8) | 5835(61.4) |
|  | **College or higher** | 905(48.5) | 565(30.3) | 794(42.5) | 304(16.3) | 1072(57.4) |
|  | **Total** | 21068 (49.3) | 11120(26.0) | 17352(40.6) | 8547(20.0) | 25122(58.8) |
|  | **p-value*** | <0 .001^a^ | < 0.001^b^ | <0 .001^c^ | <0 .001^d^ | < 0.001^e^ |
| **Hearing and speech** | **Elementary school or below** | 547(49.7) | 269 (24.4) | 402(36.5) | 229(20.8) | 635(57.7) |
|  | **Middle school** | 912(44.1) | 638(30.9) | 836(40.4) | 417(20.2) | 1184(57.3) |
|  | **High school** | 373(41.7) | 313(35.0) | 357(39.9) | 150(16.8) | 527(58.9) |
|  | **College or higher** | 92(41.6) | 73(33.0) | 92(41.6) | 35(15.8) | 125(56.6) |
|  | **Subtotal** | 1924(44.9) | 1293(30.2) | 1687(39.4) | 831(19.4) | 2471(57.7) |
|  | **p-value*** | 0.001 | < 0.001^f^ | 0.151 | 0 .047 | 0.850 |
| **Visual** | **Elementary school or below** | 845(54.6) | 394(25.5) | 599(38.7) | 380(24.5) | 919(59.4) |
|  | **Middle school** | 2395(45.8) | 1820(34.8) | 2227(42.6) | 1145(21.9) | 3227(61.7) |
|  | **High school** | 1253(44.2) | 978(34.5) | 1221(43.1) | 589(20.8) | 1801(63.6) |
|  | **College or higher** | 253(46.0) | 175(31.8) | 243(44.2) | 100(18.2) | 314(57.1) |
|  | **Subtotal** | 4746(46.7) | 3367(33.1) | 4290(42.2) | 2214(21.8) | 6261(61.6) |
|  | **p-value*** | < 0.001^j^ | < 0.001^h^ | 0.019 | 0.005 | 0.005 |
| **Physical** | **Elementary school or below** | 2384(53.2) | 772(17.2) | 1778(39.7) | 928(20.7) | 2719(60.7) |
|  | **Middle school** | 5619 (50.7) | 2771(25.0) | 4426(40.0) | 2194(19.8) | 6542(59.1) |
|  | **High school** | 2407(48.6) | 1424(28.7) | 2044(41.2) | 978(19.7) | 3040(61.3) |
|  | **College or higher** | 467(49.5) | 289(30.6) | 395(41.9) | 149(15.8) | 540(57.3) |
|  | **Subtotal** | 10877(50.7) | 5256(24.5) | 8643(40.3) | 4249(19.8) | 12841(59.9) |
|  | **p-value*** | < 0.001^i^ | < 0.001^g^ | 0.258 | 0.008 | 0.010 |
| **Intellectual** | **Elementary school or below** | 1277(49.1) | 401(15.4) | 1018(39.1) | 472(18.1) | 1262(48.5) |
|  | **Middle school** | 796(48.9) | 263(16.2) | 676(41.5) | 239(14.7) | 774(47.6) |
|  | **High school** | 107(51.4) | 31(14.9) | 91(43.8) | 35(16.8) | 99(47.6) |
|  | **College or higher** | 3(42.9) | 2(28.6) | 2(28.6) | 1(14.3) | 2(28.6) |
|  | **Subtotal** | 2183(49.1) | 697(15.7) | 1787(40.2) | 747(16.8) | 2137(48.1) |
|  | **p-value*** | 0.900 | 0.704 | 0.265 | 0.036 | 0.696 |
| **Mental** | **Elementary school or below** | 207(59.0) | 39(11.1) | 119(33.9) | 93(26.5) | 230(65.5) |
|  | **Middle school** | 533(59.3) | 188(20.9) | 353(39.3) | 201(22.4) | 510(56.7) |
|  | **High school** | 305(58.1) | 130(24.8) | 224(42.7) | 111(21.1) | 313(59.6) |
|  | **College or higher** | 84(67.7) | 23(18.5) | 51(41.1) | 16(12.9) | 76(61.3) |
|  | **Subtotal** | 1129(59.5) | 380(20.0) | 747(39.3) | 421(22.2) | 1129(59.5) |
|  | **p-value*** | 0.264 | < 0.001^k^ | 0.073 | 0.016 | 0.040 |
| **Multiple** | **Elementary school or below** | 57(45.6) | 23(18.4) | 56(44.8) | 18(14.4) | 68(54.4) |
|  | **Middle school** | 104(44.4) | 73(31.2) | 91(38.9) | 48(20.5) | 145(62.0) |
|  | **High school** | 42(45.2) | 28(30.1) | 40(43.0) | 16(17.2) | 55(59.1) |
|  | **College or higher** | 6(27.3) | 3(13.6) | 11(50.0) | 3(13.6) | 15(68.2) |
|  | **Subtotal** | 209(44.1) | 127(26.8) | 198(41.8) | 85(17.9) | 283(59.7) |
|  | **p-value*** | 0.441 | 0.026 | 0.585 | 0.488 | 0.452 |
| **Level 1** | **Elementary school or below** | 451(51.0) | 198(22.4) | 354(40.0) | 168(19.0) | 521(58.9) |
|  | **Middle school** | 787(47.8) | 458(27.8) | 688(41.8) | 356(21.6) | 1001(60.9) |
|  | **High school** | 322(45.5) | 197(27.8) | 300(42.4) | 137(19.4) | 428(60.5) |
|  | **College or higher** | 63(37.1) | 41(24.1) | 78(45.9) | 25(14.7) | 91(53.5) |
|  | **Subtotal** | 1623(47.6) | 894(26.2) | 1420(41.7) | 686(20.1) | 2041(59.9) |
|  | **p-value*** | 0.005 | 0.016 | 0.491 | 0.092 | 0.265 |
| **Level 2** | **Elementary school or below** | 547(50.3) | 177(16.3) | 404(37.2) | 215(19.8) | 586(53.9) |
|  | **Middle school** | 1372(50.9) | 673(25.0) | 1057(39.2) | 615(22.8) | 1554(57.7) |
|  | **High school** | 668(48.2) | 394(28.4) | 572(41.3) | 277(20.0) | 858(61.9) |
|  | **College or higher** | 170(53.8) | 75(23.7) | 133(42.1) | 49(15.5) | 178(56.3) |
|  | **Subtotal** | 2757(50.3) | 1319(24.1) | 2166(39.5) | 1156(21.1) | 3176(57.9) |
|  | **p-value*** | 0.222 | < 0.001^l^ | 0.153 | 0.005 | 0.001 |
| **Level 3** | **Elementary school or below** | 1620(52.4) | 491(15.9) | 1184(38.3) | 602(19.5) | 1787(57.8) |
|  | **Middle school** | 3006(49.0) | 1444(23.5) | 2482(40.4) | 1063(17.3) | 3523(57.4) |
|  | **High school** | 1207(46.7) | 704(27.2) | 1071(41.4) | 496(19.2) | 1534(59.3) |
|  | **College or higher** | 237(47.6) | 134(26.9) | 214(43.0) | 76(15.3) | 294(59.0) |
|  | **Subtotal** | 6070(49.3) | 2773(22.5) | 4951(40.2) | 2237(18.2) | 7138(58.0) |
|  | **p-value*** | < 0.001^m^ | < 0.001^n^ | 0.046 | 0.011 | 0.387 |
| **Level 4** | **Elementary school or below** | 2699(52.5) | 1032(20.1) | 2030(39.5) | 1135(22.1) | 2939(57.1) |
|  | **Middle school** | 5194(48.8) | 3178(29.8) | 4382(41.1) | 2210(20.7) | 6304(59.2) |
|  | **High school** | 2290(47.4) | 1609(33.3) | 2034(42.1) | 969(20.1) | 3015(62.4) |
|  | **College or higher** | 435(49.3) | 315(35.7) | 369(41.8) | 154(17.4) | 509(57.6) |
|  | **Subtotal** | 10618(49.4) | 6134(28.5) | 8815(41.0) | 4468(20.8) | 12767(59.4) |
|  | **p-value*** | < 0.001^o^ | < 0.001^p^ | 0.051 | 0.005 | < 0.001^q^ |

*χ2 test and Fisher’s exact test were conducted to compare the prevalence of each health outcome in different education levels.

^a^ p = 8.08620222642061E-11 ^b^ p = 4.12037312310817E-92 ^c^ p = 0.000107210522360043 ^d^ p = 0.000144998989757823 ^e^ p = 1.62261094666859E-08

^f^ p = 3.12302572411109E-06 ^g^ p =2.94192672999227E-10 ^h^ p = 5.51669620111951E-11 ^i^ p = 0.000111660991424272 ^j^ p = 2.04694838804187E-42

^k^ p =0.0000127694131019277 ^l^ p = 3.46362411394606E-11 ^m^ p =0.000185619627816675 ^n^ p = 7.79213264752363E-26 ^o^ p =2.40217947128789E-06

^p^ p = 2.26450131270884E-57 ^q^ p = 1.02180388285092E-06
