# Supplementary material for: Association between education and health outcomes among adults with disabilities: evidence from Shanghai, China
Source: PeerJ. 2019 Feb 19;7:e6382. doi: 10.7717/peerj.6382 (PMC6385680; doi:10.7717/peerj.6382)
Supplement: Table S3 — 1Adjusted for gender, age, hukou, marital status, disability type, and disability severity. 2Adjusted for gender, age, hukou, marital status and disability severity. OR, odds ratio; CI, confidence interval a, p = 1.82610338670946E–09 b, p = 3.59421532803415E–65 c, p = 5.21541397814597E–12 d, p = 5.34560735401499E–73 e, p = 0.000485652226983674 f, p = 0.0000228939475230585 g, p = 1.46498546264525E–38 h, p = 3.36865066099832E–07 i, p = 5.65373183403489E–07 j, p = 2.05001733219406E–08 k, p = 0.0000069875277559385 l, p = 2.0130374120598E–08 m, p = 5.73649683295901E–16 n, p = 9.50661598792444E–08 o, p = 9.00383872834067E–14 p, p = 0.000111084046409749 q, p = 3.22739499284614E–08 r, p = 1.23882486591996E–44 s, p = 0.0000848185284451649 t, p = 2.57456171857619E–56 u, p = 2.12628072004128E–33 v, p = 0.0000379636759741926 w, p = 9.13903589573368E–07. [file peerj-07-6382-s005.docx]

**Table 3. Results of Logistic Regressions of Health Outcomes with Education Level across Disability Types**.

|  | **BMI ≥ 24** | | |  |  | **Haemorrhoid** | | |  |  | **Fatty liver** | | |  |  | **High blood glucose** | | |  |  | **High blood lipid** | | |  |
| --- | --- | --- | --- | --- | --- | --- | --- | --- | --- | --- | --- | --- | --- | --- | --- | --- | --- | --- | --- | --- | --- | --- | --- | --- |
|  | **OR** | **[95% CI]** | | **p** |  | **OR** | **[95% CI]** | | **p** |  | **OR** | **[95% CI]** | | **p** |  | **OR** | **[95% CI]** | | **p** |  | **OR** | **[95% CI]** | | **p** |
| ***Total*** |  |  | |  |  |  |  | |  |  |  |  | |  |  |  |  | |  |  |  |  | |  |
| ***Education*^1^** |  |  |  |  |  |  |  |  |  |  |  |  |  |  |  |  |  |  |  |  |  |  |  |  |
| Elementary school or below | Reference | | | |  | Reference | | | |  | Reference | | | |  | Reference | | | |  | Reference | | | |
| Middle school | 0.85 | 0.81 | 0.90 | <0 .001^a^ |  | 1.76 | 1.65 | 1.88 | <0 .001^b^ |  | 1.08 | 1.02 | 1.14 | 0.005 |  | 0.95 | 0.89 | 1.01 | 0.087 |  | 1.01 | 0.96 | 1.07 | 0.655 |
| High school | 0.80 | 0.75 | 0.85 | <0 .001^c^ |  | 2.03 | 1.88 | 2.19 | <0 .001^d^ |  | 1.12 | 1.05 | 1.20 | <0 .001^e^ |  | 0.91 | 0.84 | 0.98 | 0.015 |  | 1.08 | 1.01 | 1.15 | 0.018 |
| College or higher | 0.80 | 0.72 | 0.89 | <0 .001^f^ |  | 2.21 | 1.96 | 2.49 | <0 .001^g^ |  | 1.15 | 1.03 | 1.27 | 0.009 |  | 0.70 | 0.61 | 0.80 | <0 .001^h^ |  | 1.06 | 0.96 | 1.18 | 0.268 |
| ***Hearing & Speech*** |  |  |  |  |  |  |  |  |  |  |  |  |  |  |  |  |  |  |  |  |  |  |  |  |
| ***Education*^2^** |  |  |  |  |  |  |  |  |  |  |  |  |  |  |  |  |  |  |  |  |  |  |  |  |
| Elementary school or below | Reference | | | |  | Reference | | | |  | Reference | | | |  | Reference | | | |  | Reference | | | |
| Middle school | 0.81 | 0.69 | 0.94 | 0.007 |  | 1.60 | 1.33 | 1.92 | <0 .001^i^ |  | 1.14 | 0.97 | 1.34 | 0.103 |  | 1.02 | 0.84 | 1.24 | 0.840 |  | 1.01 | 0.86 | 1.18 | 0.951 |
| High school | 0.73 | 0.60 | 0.89 | 0.002 |  | 1.89 | 1.51 | 2.36 | <0 .001^j^ |  | 1.10 | 0.90 | 1.34 | 0.351 |  | 0.77 | 0.60 | 0.99 | 0.043 |  | 1.07 | 0.88 | 1.31 | 0.485 |
| College or higher | 0.69 | 0.51 | 0.95 | 0.021 |  | 2.20 | 1.56 | 3.10 | <0 .001^k^ |  | 1.24 | 0.91 | 1.69 | 0.169 |  | 0.78 | 0.52 | 1.17 | 0.229 |  | 1.30 | 0.95 | 1.78 | 0.106 |
| ***Visual Disability*** |  |  |  |  |  |  |  |  |  |  |  |  |  |  |  |  |  |  |  |  |  |  |  |  |
| ***Education*^2^** |  |  |  |  |  |  |  |  |  |  |  |  |  |  |  |  |  |  |  |  |  |  |  |  |
| Elementary school or below | Reference | | | |  | Reference | | | |  | Reference | | | |  | Reference | | | |  | Reference | | | |
| Middle school | 0.71 | 0.63 | 0.80 | <0 .001^l^ |  | 1.79 | 1.55 | 2.06 | <0 .001^m^ |  | 1.19 | 1.05 | 1.34 | 0.007 |  | 0.91 | 0.79 | 1.05 | 0.192 |  | 1.10 | 0.97 | 1.25 | 0.129 |
| High school | 0.69 | 0.60 | 0.79 | <0 .001^n^ |  | 1.82 | 1.56 | 2.13 | <0 .001^o^ |  | 1.22 | 1.06 | 1.40 | 0.005 |  | 0.88 | 0.74 | 1.03 | 0.115 |  | 1.16 | 1.01 | 1.34 | 0.040 |
| College or higher | 0.67 | 0.55 | 0.82 | <0 .001^p^ |  | 1.92 | 1.52 | 2.41 | <0 .001^q^ |  | 1.27 | 1.03 | 1.55 | 0.024 |  | 0.68 | 0.53 | 0.88 | 0.003 |  | 1.06 | 0.86 | 1.31 | 0.561 |
| ***Physical Disability*** |  |  |  |  |  |  |  |  |  |  |  |  |  |  |  |  |  |  |  |  |  |  |  |  |
| ***Education*^2^** |  |  |  |  |  |  |  |  |  |  |  |  |  |  |  |  |  |  |  |  |  |  |  |  |
| Elementary school or below | Reference | | | |  | Reference | | | |  | Reference | | | |  | Reference | | | |  | Reference | | | |
| Middle school | 0.90 | 0.83 | 0.96 | 0.004 |  | 1.99 | 1.81 | 2.19 | <0 .001^r^ |  | 1.01 | 0.93 | 1.09 | 0.866 |  | 1.00 | 0.91 | 1.10 | 0.989 |  | 1.01 | 0.94 | 1.09 | 0.798 |
| High school | 0.84 | 0.77 | 0.91 | <0 .001^s^ |  | 2.46 | 2.20 | 2.75 | <0 .001^t^ |  | 1.05 | 0.96 | 1.15 | 0.257 |  | 0.99 | 0.89 | 1.11 | 0.935 |  | 1.09 | 1.00 | 1.20 | 0.059 |
| College or higher | 0.86 | 0.74 | 0.99 | 0.041 |  | 2.84 | 2.40 | 3.36 | <0 .001^u^ |  | 1.06 | 0.91 | 1.23 | 0.448 |  | 0.73 | 0.60 | 0.89 | 0.002 |  | 1.07 | 0.92 | 1.24 | 0.407 |
| ***Intellectual Disability*** | |  |  |  |  |  |  |  |  |  |  |  |  |  |  |  |  |  |  |  |  |  |  |  |
| ***Education*^2^** |  |  |  |  |  |  |  |  |  |  |  |  |  |  |  |  |  |  |  |  |  |  |  |  |
| Elementary school or below | Reference | | | |  | Reference | | | |  | Reference | | | |  | Reference | | | |  | Reference | | | |
| Middle school | 0.97 | 0.85 | 1.10 | 0.606 |  | 1.11 | 0.93 | 1.33 | 0.248 |  | 1.13 | 0.99 | 1.29 | 0.063 |  | 0.77 | 0.65 | 0.92 | 0.004 |  | 1.03 | 0.90 | 1.17 | 0.694 |
| High school | 1.07 | 0.80 | 1.43 | 0.635 |  | 0.93 | 0.61 | 1.40 | 0.716 |  | 1.24 | 0.93 | 1.66 | 0.148 |  | 0.83 | 0.56 | 1.22 | 0.335 |  | 0.96 | 0.72 | 1.29 | 0.803 |
| College or higher | 0.75 | 0.17 | 3.36 | 0.703 |  | 2.90 | 0.52 | 16.22 | 0.225 |  | 0.66 | 0.13 | 3.43 | 0.623 |  | 1.01 | 0.12 | 8.57 | 0.996 |  | 0.59 | 0.11 | 3.10 | 0.535 |
| ***Mental Disability*** |  |  |  |  |  |  |  |  |  |  |  |  |  |  |  |  |  |  |  |  |  |  |  |  |
| ***Education*^2^** |  |  |  |  |  |  |  |  |  |  |  |  |  |  |  |  |  |  |  |  |  |  |  |  |
| Elementary school or below | Reference | | | |  | Reference | | | |  | Reference | | | |  | Reference | | | |  | Reference | | | |
| Middle school | 0.96 | 0.73 | 1.25 | 0.736 |  | 2.31 | 1.55 | 3.43 | <0 .001^v^ |  | 1.26 | 0.96 | 1.66 | 0.097 |  | 0.91 | 0.67 | 1.24 | 0.546 |  | 0.72 | 0.55 | 0.95 | 0.020 |
| High school | 0.88 | 0.65 | 1.20 | 0.412 |  | 3.01 | 1.94 | 4.68 | <0 .001^w^ |  | 1.43 | 1.05 | 1.96 | 0.025 |  | 0.88 | 0.61 | 1.26 | 0.486 |  | 0.84 | 0.62 | 1.15 | 0.290 |
| College or higher | 1.23 | 0.77 | 1.96 | 0.381 |  | 2.35 | 1.27 | 4.35 | 0.007 |  | 1.38 | 0.88 | 2.17 | 0.165 |  | 0.54 | 0.29 | 1.01 | 0.053 |  | 0.99 | 0.63 | 1.56 | 0.971 |

| Multiple Disabilities |  |  |  |  |  |  |  |  |  |  |  |  |  |  |  |  |  |  |  |  |  |  |  |  |
| --- | --- | --- | --- | --- | --- | --- | --- | --- | --- | --- | --- | --- | --- | --- | --- | --- | --- | --- | --- | --- | --- | --- | --- | --- |
| ***Education*^2^** |  |  |  |  |  |  |  |  |  |  |  |  |  |  |  |  |  |  |  |  |  |  |  |  |
| Elementary school or below | Reference | | | |  | Reference | | | |  | Reference | | | |  | Reference | | | |  | Reference | | | |
| Middle school | 0.92 | 0.57 | 1.50 | 0.739 |  | 1.62 | 0.88 | 2.99 | 0.123 |  | 0.83 | 0.51 | 1.35 | 0.454 |  | 1.74 | 0.88 | 3.44 | 0.111 |  | 1.32 | 0.80 | 2.18 | 0.274 |
| High school | 0.88 | 0.48 | 1.63 | 0.693 |  | 1.58 | 0.75 | 3.34 | 0.229 |  | 1.07 | 0.57 | 1.98 | 0.840 |  | 1.22 | 0.53 | 2.82 | 0.643 |  | 1.09 | 0.58 | 2.06 | 0.781 |
| College or higher | 0.43 | 0.16 | 1.21 | 0.112 |  | 0.63 | 0.16 | 2.45 | 0.509 |  | 1.49 | 0.58 | 3.82 | 0.411 |  | 0.84 | 0.21 | 3.28 | 0.801 |  | 1.58 | 0.57 | 4.33 | 0.377 |

^1^ Adjusted for gender, age, *hukou*, marital status, disability type, and disability severity.

^2^ Adjusted for gender, age, *hukou*, marital status and disability severity.

OR, odds ratio; CI, confidence interval

^a^p=1.82610338670946E-09 ^b^p=3.59421532803415E-65 ^c^p=5.21541397814597E-12 ^d^p=5.34560735401499E-73 ^e^p= 0.000485652226983674

^f^ p=0.0000228939475230585 ^g^p=1.46498546264525E-38 ^h^ p=3.36865066099832E-07 ^i^ p=5.65373183403489E-07 ^j^p=2.05001733219406E-08

^k^ p=0.0000069875277559385 ^l^p= 2.0130374120598E-08 ^m^ p=5.73649683295901E-16 ^n^ p=9.50661598792444E-08 ^o^p=9.00383872834067E-14

^p^ p=0.000111084046409749 ^q^ p=3.22739499284614E-08 ^r^ p=1.23882486591996E-44 ^s^p= 0.0000848185284451649

^t^ p=2.57456171857619E-56 ^u^ p=2.12628072004128E-33 ^v^ p=0.0000379636759741926 ^w^p= 9.13903589573368E-07
